# Determinants of cervical cancer screening service utilization among women attending healthcare services in Amhara region referral hospitals: a case–control study

**DOI:** 10.1186/s12905-022-02071-8

**Published:** 2022-12-02

**Authors:** Temesgen Dessalegne Legasu, Kibir Temesgen, Zenebe Tefera Ayele, Moges Sisay Chekole, Fekade Demeke Bayou, Jemberu Chane Fetene, Abebe Tadesse Tibebu, Birhan Tsegaw Taye, Mohammed Ahmed Ali

**Affiliations:** 1 Department of Midwifery, College of Medicine and Health Sciences, Jigjiga University, P. Box 1020, Jigjiga, Ethiopia; 2 Department of Clinical Midwifery, School of Nursing &Midwifery, College of Medicine and Health Sciences, Wollo University, Wollo, Ethiopia; 3 School of Nursing and Midwifery, Asrat Woldeyes Health Science Campus, Debre Berhan University, Debre Berhan, Ethiopia; 4 Department of Epidemiology, College of Medicine and Health Sciences, Jigjiga University, Jigjiga, Ethiopia

**Keywords:** Cervical cancer screening, Determinants, Case–control, Utilization, Ethiopia

## Abstract

**Background:**

Cervical cancer is the second leading cause of cancer-related death and one of the top 20 causes of death among women in Ethiopia. Cervical cancer screening service has a vital value to reduce morbidity and mortality. Even though cervical cancer screening service utilization in Ethiopia is unacceptably low, its determinant factors were not well studied in the study area. Hence, this study was aimed at filling this information gap. This study aimed to identify determinants of cervical cancer screening service utilization among women attending healthcare services in Amhara region referral hospitals, Ethiopia.

**Methods:**

Hospital-based case–control study was conducted among 441 women (147 cases and 294 controls) from May to July 2021. Cases were included consecutively and controls were selected using a systematic random sampling technique from the randomly selected hospitals. A pretested interviewer-administered questionnaire was used to collect the data from respondents. The data were entered into Epi data version 4.6 and exported to SPSS version 25 for analysis. Bivariable and multivariable logistic regression analysis was employed. Adjusted odds ratio with its 95% confidence interval and *p* value < 0.05 were used to estimate the strength and significance of the association.

**Result:**

A total of 147 cases and 294 controls were enrolled in this study. Women with 30–39 years-old [AOR = 2.3; 95% CI 1.21, 4.68] and 40–49 years-old [AOR = 4.4 95% CI 1.97, 10.12], urban residence [AOR = 2.6; 95% CI 1.36, 5.21], secondary education [AOR = 4.4; 95% CI 2.18, 8.87] and diploma and above [AOR = 2.0; 95% CI: 1.05, 4.59], ever gave birth [AOR = 9.4; 95% CI 4.92, 18.26], having multiple sexual partners [AOR = 2.8; 95% CI 1.60, 5.03], good knowledge towards cervical cancer screening [AOR = 3.6; 95% CI 2.07, 6.43] and positive attitude on cervical cancer screening [AOR = 2.0, 95% CI 1.20, 3.70] were significant determinants of cervical cancer screening service utilization.

**Conclusion:**

In this study, age (30–39 and 40–49), urban residence, secondary education, ever gave birth, good knowledge of cervical cancer screening, positive attitude towards cervical cancer screening, and having multiple sexual partners were significant determinants of cervical cancer screening service utilization. There is a need to strengthen the policy and health education on safe sexual practices and healthy lifestyles through information dissemination and communication to scale up screening service utilization.

## Introduction

Cervical cancer is a malignant neoplasm from cells originating in the cervix uteri, and sometimes it can be asymptomatic in the early stages of the disease’s progress [1]. It is caused by human papillomavirus (HPV) infection, which is transmitted through sexual intercourse and infects the areas around the cervix, anus, mouth, and throat [2]. At the early-stage cervical cancer may be manifested with watery, blood-tinged vaginal discharge. Intermittent vaginal bleeding that follows coitus or douching may also be noted. As the malignancy enlarges, bleeding typically intensifies and occasionally follows uncontrolled hemorrhage [1].

Cervical cancer is still the most common female cancer globally and the leading cause of cancer deaths among females in Africa**.** It commonly occurs in women aged ranged from 25 to 65 years [3]. Among female cancers, cervical cancer is a leading cause of morbidity and mortality throughout the world, especially among women in Africa [4]. Of all the cancers, cervical cancer is the only one that has both an effective vaccine and screening program that can prevent disease and death [5].

Cervical cancer screening service is aimed at early detection and treatment of the disease [6]. In developed countries, the establishment of national screening programs with a population-based invitation of eligible women and a recall system for screen positives resulted in the prevention of almost 80% of cervical cancer [7]. Cervical cancer screening service utilization is recommended for effective methods for the prevention and early detection of cervical cancer [8]. Thus, evidence support the possibility to use specific biomarkers to identify early-stage cervical cancer (precancerous cervical lesion) which offer a better prognosis to the patients [9–11]. However, global evidence shows that the utilization of screening for prevention is very poor in developing countries. Lack of knowledge about cervical screening behavior among lower socio-economic status was found to be the primary barrier to the utilization of cervical cancer screening services [12, 13].

The introduction of Pap smear screening in western states reduce the incidence and mortality of cervical cancer. However cervical cancer still remains the main cause of death from cervical cancer in less developed settings where screening service utilization is scant or non-existent screening programs [10]. Recent evidence also showed that high risk Human Papilloma Virus (HPV) negative high grade abnormal type within a tissue of cervix have more improved results than patients with documented high risk HPV infection [10, 14]. Primary conization before radical hysterectomy for early-stage cervical cancer has also a potential protective role for patients with International Federation of Gynecology and Obstetrics (IFGO) stage IB1 cervical cancer [15, 16].

Cervical cancer is the fourth most common cancer accounting for 6.6% among women worldwide [17]. About 85% of the global burden and 87% of mortalities occur in developing countries. Of these, East African region is the top bookkeeping with an age-standardized incidence of 42.7/100,000 population and a mortality rate of 27.6/100,000 deaths [8]. In Ethiopia, it is the second most common female cancer with an incidence rate of 18.9/100,000 in 2018 [18]. Based on this public health significance, Ethiopia designed a strategic goal to reduce the incidence of cervical cancer and mortality by 15% by increasing the vaccination coverage against HPV [19]. To achieve its ambitious goals, cervical cancer screening services were being provided free for all eligible women since 2016 [20]. Despite the availability of guidelines for cervical cancer prevention and control; screening service was not fully utilized in all healthcare facilities [21]. The problem arose from inadequate knowledge about cervical cancer screening, limited treatment facilities,and too few government, and other stakeholders’ interventions [22]. As a result, most of the women in the country visit health institutions with the advanced stage of cervical cancer. Cervical cancer is the leading cause of cancer mortality among Ethiopian women over the age of 30 [23].

Cervical cancer screening has been shown as the most effective way to decrease cancer mortality among all women [24]. In Ethiopia, only 0.6% of all women, 1.6% of urban, and 0.4% of rural women aged 18–69 years screened every three years [25, 26]. The major factors associated with low cervical cancer screening were inadequate knowledge about the disease process, available screening methods, and client’s negative attitudes toward the procedure [17, 27, 28]. In Ethiopia, screening is usually conducted only when a woman seeks medical care for other reasons [29]. Regardless of the evidence that universal screening is important, women without symptoms are not routinely screened in many areas of Ethiopia [30]. Despite low cervical cancer screening service utilization in the country, evidence about the determinants of cervical cancer screening service utilization in Ethiopia, particularly in the study setting were scarce. Therefore, this study identified the determinants of cervical cancer screening service utilization among women attending health care services in Amhara region referral hospitals, in northwest Ethiopia.

## Method and materials

### Study setting and period

The study was conducted in referral hospitals of Amhara regional state, Northeastern and North-central part of Ethiopia. Amhara region is the second most populous region in Ethiopia. It has 12 zones, 3 city administrations, and 180 woredas. According to the Ethiopian Central Statistics Agency population projection, the region has a total population of 21.5 million people with a fifty-fifty numerical split between the sexes. Currently, the region has 84 hospitals (6 referrals, 14 general, and 64 primaries), 847 health centers, and 3,342 health posts [31]. The referral hospitals are the University of Gondar comprehensive and specialized referral hospital, Felege Hiwot comprehensive and specialized referral hospital, Debre Markos comprehensive and specialized referral hospital, Debre Berhan comprehensive and specialized referral hospital, Tibebe Gion, and Dessie comprehensive and specialized referral hospital. These referral hospitals are providing a broad range of services to outpatient, hospitalized patients and emergency cases including cervical cancer prevention services, and treatments. Each hospital is assumed to be serving 5.2 million people, has 200–400 beds, and around 1488 precancerous cases annually [32]. A hospital-based unmatched case–control study was conducted from May 1 to July 30, 2021.

### Source and study population

All women attending health care services in Amhara region referral hospitals were the source population. All women attending health care services in the selected referral hospitals of the Amhara region during the study period were the study population. (Cases: were women who were newly utilizing screening services for cervical cancer during the study period. Controls: were women who visited the gynecologic outpatient department during the study period for other health services (other than cervical cancer screening service).

### Eligibility criteria

For cases; all women who visited the gynecologic OPD for cervical cancer screening in the selected Amhara region referral hospitals during the study period were included in this study. For controls; all women who visited the gynecologic OPD other than cervical cancer screening in the selected Amhara region referral hospitals during the study period were included in this study. Women who were unable to hear and unconscious at the time of the study and those who were linked by health care professionals for cervical cancer screened by indication were excluded from the study.

### Sample size determination

The sample size for the study was computed using Epi info version 7.2.3 software, by considering the following assumptions; 95% level of significance, 80% of power, the ratio of cases to controls 1:2, the proportion of exposure among controls 38.8%, Odds ratio 2.15, the proportion of cases with exposure 57.7% from a previous similar study conductedin Mekelle [33] and design effect of 1.5. Thus, the sample size for this study was 267, by considering a 10% non-response rate and design effect, the final sample size was 294*1.5 design effect = 441 individuals (147 cases and 294 controls).

### Sampling procedure

From a total of six (6) referral hospitals in the Amhara region, three (3) of them were selected by simple random sampling technique (lottery method). The selected referral hospitals were Debre Birhan referral hospital, TibebeGion referral hospital, and Dessie referral hospital. The total sample size was allocated to each selected referral hospital by using probability proportionate to the average monthly client flow, reviewed from the registration book. Cases were selected consecutively, i.e. by including all women screened for cervical cancer during the data collection period until the required sample size is achieved and controls were selected using a systematic random sampling technique. The first respondent from the control was selected randomly from all women who visit the gynecologic outpatient department and the next participant was included after every fourth interval.

### Study variables

*Dependent Variable* Cervical cancer screening utilization (Yes/No).

*Independent Variables* included socio-demographic, and economic characteristics (age, marital status, level of education, occupational status, family monthly income and place of residence), reproductive, sexual behavior, behavioral and obstetric related factors (contraceptive utilization, Parity, Smoking status, history of STI and Multiple sexual partners), Knowledge for cervical cancer screening and Attitudes for cervical cancer screening.

### Operational definitions

*Knowledge about Cervical cancer screening* the study participants were asked 12 knowledge- assessing questions. These items comprise "Yes" or "No" and multiple response options. One point was given for all the correct answers, while zero points were for incorrect answers. Then, based on the summative score, women's knowledge was composed (of good knowledge which was coded as "1" and poor knowledge which was coded as "0"). Thus, participants with a score of mean and above were considered as good knowledge otherwise poor knowledge [34].

*Positive attitude towards cervical cancer screening* Women's attitude towards cervical cancer screening utilization was measured using 11 attitude assessing questions. Each question has five points Likert scale (1 = strongly disagree, 2 = disagree, 3 = neutral, 4 = agree, 5 = strongly agree). Women who scored mean and above value were considered to have a positive attitude and those who scored less than the mean value were considered a negative attitude [34].

### Data collection tools and procedures

Data were collected by face-to-face interviews using structured, pretested questionnaires adapted from different kinds of literature [4, 33, 35, 36]. The questionnaire comprises questions on socio-demographic and economic status, attitude, knowledge, reproductive, behavioral, and sexual behavior characteristics of study participants. The questionnaire was first prepared in English and translated to the local language (i.e. Amharic) then back to English to keep its consistency. Data were collected by three trained BSc. degree midwives who are experienced in cervical cancer screening services and working at the Gynecologic clinic during the time of the data collection period. The data collection process was supervised by three BSc midwives who had research skills.

### Data quality control

To make sure the quality of data, one-day training was provided for the data collectors and supervisors in each referral hospital about techniques of data collection. Close supervision was made during the whole period of data collection and checked each questionnaire for completeness and consistency on a daily basis. The questionnaire was pre-tested on 5% [22] of the study population at Felege Hiwot referral hospital (which was not included in the actual data collection) to check the understandability and reliability of the questionnaires.

### Data processing and analysis

The collected data were checked for completeness and consistency and then each questionnaire was coded and entered into Epi Data version 4.6.0 software and exported to SPSS version 25.0 for analysis. The Hosmer–Lemeshow test was checked to evaluate whether the assumption for binary logistic regression was fulfilled or to evaluate model fitness. The test result revealed a P-value of 0.6, which indicates that the assumption is fulfilled and the model is fitted with data. Multicollinearity was checked using the variance inflation factor (VIF) and the output indicated that there was no multicollinearity. A bivariable logistic regression was done and variables with p-value < 0.2 in a bivariable analysis were entered into multivariable regression analysis to identify determinant factors. A variable with a P-value of < 0.05 in the multivariable analysis was considered a measure of statistically significant determinants. Adjusted odds ratio with its 95% confidence interval was used to measure the strength of association between variables. The results were described and presented by tables, graphs, and narrative descriptions.

## Results

### Socio-demographic characteristics of the participants

A total of 441 women (147 cases and 294 controls) participated in the study, giving a response rate of 100%. Nearly half (49.0%) of the controls and 44.2% of the cases were between the age categories of 21–29 and 30–39 years respectively. The mean and standard deviation of the age of the cases and controls was 28.7 ± 7.6 and 25.5 ± 5.9 years, respectively. One hundred fourteen (77.6%) of cases and two hundred fifty- five (86.7%) of controls were married. Concerning the occupational status of participants, 25 (17.0%) of cases and forty-two (14.3%) of controls were housewives. Regarding educational status of the participants, 65 (44.2%) of cases and 209 (71.1%) of controls had no formal education, whereas 33 (22.4%) of cases and 41 (13.9%) of controls had attained higher education. More than half (55.8%) of cases and the majority (89.1%) of controls were rural residents (Table 1).

**Table 1:** Socio-demographic characteristics of women attending health care services in Amara region referral hospitals, Northwest Ethiopia, 2021G.C (n = 441)

Variables	Cases n (%)	Controls n (%)
Age (in year n = 441)		
21–29	30 (20.4)	144 (49.0)
30–39	65 (44.2)	118 (40.1)
40–49	52 (35.4)	32 (10.9)
Marital status (n = 441)		
Single	8 (5.4)	18 (6.0)
Married	114 (77.6)	255 (87.0)
Widowed	7 (4.8)	3 (1.0)
Divorced	18 (12.2)	18 (6.0)
Educational status of participant (n = 441)		
No formal education	65 (44.2)	209 (71.1)
Completed secondary education	49 (33.3)	44 (15.0)
Diploma and above	33 (22.4)	41 (13.9)
Educational status of husband (n = 369)		
No formal education	55 (48.2)	134 (52.6)
Completed secondary education	30 (26.3)	48 (18.8)
Diploma and above	29 (25.5)	73 (28.6)
Occupation of participant (n = 441)		
House wife	25 (17.0)	42 (14.3)
Private	9 (6.1)	52 (7.7)
Gov’t	113 (76.9)	200 (68.0)
Occupation of husband (n = 369)		
Farmer	55 (48.2)	93 (36.5)
Private	17 (15.0)	51 (20.0)
Gov’t	42 (36.8)	111 (43.5)
Average monthly income in ETB		
≤ 1000	15 (10.2)	38 (12.9)
1001–2000	19 (13.0)	43 (14.7)
≥ 2000	70 (47.6)	99 (33.6)
Unknown	43 (29.2)	114 (38.8)
Residence (n = 441)		
Urban	65 (44.2)	32 (10.9)
Rural	82 (55.8)	262 (89.1)

*ETB* Ethiopian Birr

### Reproductive health-related characteristics of the participants

The majority of the study participants (88.4% of the cases and 89.4% of the controls) had their first sex at age of ≤ 18 years old. On the other hand, 128 (87.1%) of the cases and 127 (43.2%) of the controls had ever given birth. Sixty-four (43.5%) of the cases and 140 (47.6%) of the controls had ever used contraceptives. The Majority (83.0%) of the cases and (79.9%) of the controls had ever tested for HIV. Regarding the number of sexual partners, the majority (78.2%) of the cases and 135 (45.9%) of the controls had two or more sexual partners (Table 2).

**Table 2:** Reproductive and individual health related characteristics of women attending health care services in Amhara region referral hospitals, Northwest Ethiopia, 2021G.C (n = 441)

Variable	Case	Control
Age of first sex (n = 441)		
< 18	130 (88.4)	254 (89.4)
≥ 18	17 (11.6)	40 (10.6)
Ever give birth (n = 441)		
Yes	128 (87.1)	127 (43.2)
No	19 (12.9)	167 (56.8)
No of children (n = 255)		
< 3	86 (67.2)	91 (71.1)
≥ 3	42 (32.8)	36 (28.9)
Ever use contraceptive (n = 441)		
Yes	64 (43.5)	140 (47.6)
No	83 (56.5)	154 (52.4)
Oral contraceptive		
Yes	17 (35.4)	32 (48.5)
No	31 (64.6)	36 (51.5)
Injectable		
Yes	30 (57.7)	49 (46.2)
No	22 (42.3)	57 (53.8)
Implanon		
Yes	17 (48.6)	27 (46.6)
No	18 (51.4)	31 (53.4)
IUCD		
Yes	0	32 (51.6)
No	12 (100.)	30 (48.4)
Duration of contraceptive use (n = 204)		
< 5 year	53 (83.0)	72 (51.4)
≥ 5 year	11 (17.0)	68 (48.6)
Current use of contraceptive (n = 441)		
Yes	28 (19.0)	69 (23.5)
No	119 (81.0)	225 (76.5)
Oral contraceptive		
Yes	4 (8.3)	11 (11.7)
No	44 (91.7)	83 (88.3)
Injectable		
Yes	19 (28.4)	44 (35.8)
No	48 (71.6)	79 (64.2)
Implanon		
Yes	5 (15.6)	14 (18.2)
No	27 (84.4)	63 (81.8)
Smoking		
Yes	5 (3.4)	0
No	142 (96.6)	294 (100.0)
History of STD		
Yes	2 (1.4)	1 (0.3)
No	145 (98.6)	293 (99.7)
HIV tested (n = 441)		
Yes	122 (83.0)	124 (42.2)
No	25 (17.0)	170 (57.8)
HIV result (n = 246)		
Positive	53 (43.4)	9 (7.3)
Negative	69 (56.6)	115 (92.7)
Know someone with cervical cancer		
Yes	7 (4.8)	0
No	140 (95.2)	294 (100.0)
Family history of cervical cancer		
Yes	4 (2.7)	3 (1.0)
No	143 (97.3)	291 (99.0)
No of sexual partner (n = 441)		
≥ 2	115 (78.2)	135 (45.9)
1	32 (21.8)	159 (54.1)
Does your partner have other partners		
Yes	79 (53.7)	66 (22.4)
No	68 (46.3)	228 (77.6)

*IUCD* intra uterine contraceptive device, *HIV* human immunodeficiency virus, *STD* sexually transmitted disease

### Knowledge and attitude on cervical cancer and its screening

A majority (68.7%) of the cases and 112 (38.1%) of the controls had good knowledge about cervical cancer screening services. More than half (60.5%) of cases and 133 (45.2%) of controls had a positive attitude towards cervical cancer screening services (Table 3).

**Table 3:** Knowledge and attitude towards cervical cancer screening services women attending health care services in Amhara region referral hospital North West Ethiopia, 2021G.C (n = 441)

Variable	Case n (%)	Control n (%)
Knowledge (n = 441)		
Good knowledge	101 (68.7)	112 (38.1)
Poor knowledge	46 (31.3)	182 (61.9)
Attitudes towards cervical cancer screening services (n = 441)		
Negative attitude	58 (39.5)	161 (54.8)
Positive attitude	89 (60.5)	133 (45.2)

### Determinants of cervical cancer screening service utilization

Bi-variable and multivariable logistic regression analysis was carried out to see the association between explanatory variables and the outcome of the interest. Variables with a p-value < 0.2 on bi-variable logistic regression were further run under Multivariable logistic regression. Accordingly, variables including age group (30–39, and 40–49 years), educational status (completed secondary, diploma and above), residing in urban residence, ever gave birth, having multiple sexual partners, good attitude and knowledge of cervical cancer were independent predictors of cervical cancer screening service utilization at a p-value of < 0.05 during multivariable analysis.

This study revealed that women being in the age group of 30–39 and 40–49 years were two times and four times more likely to utilize cervical cancer screening services than those who were in the age group of 21–29 years (AOR = 2.3; 95% CI 1.21, 4.68) and (AOR = 4.4; 95% CI 1.97, 10.12 respectively). Regarding educational status, women who completed secondary education and diploma and above were four times and two times more likely to utilize CCA screening services than those who had no formal education with (AOR = 4.4; 95% CI 2.18, 8.87) and (AOR = 2.0; 95% CI 1.05, 4.59 respectively). Participants who had ever given birth were nine times more likely to utilize cervical cancer screening services (AOR = 9.4, 95% CI 4.92, 18.26) than their counterparts. Women having multiple sexual partners were nearly three times more likely to utilize cervical cancer screening services (AOR = 2.8; 95% CI 1.60, 5.03) than their counterparts. On the other hand, participants with a positive attitude towards cervical cancer screening were two times more likely to utilize cervical cancer screening services than their counterparts (AOR = 2.0; 95% CI 1.20, 3.70). Women who live in urban areas were 2.6 times more likely to utilize cervical cancer screening services than their counterparts (AOR = 2.6; 95% CI 1.36, 5.21). Women who have good Knowledge were four times more likely to utilize cervical cancer screening services than their counterparts (AOR = 3.6; 95% CI 2.07, 6.43) (Table 4).

**Table 4:** Bivariable and multivariable analysis of selected variables on determinants of CCA screening service utilization women attending health care service in Amara region referral hospitals, Northwest Ethiopia, 2021 G.C (n = 441)

Variable	Case n (%)	Control n (%)	COR (95%CI)	AOR (95%CI)	p-value
Age (in year)					
21–29	30 (20.4)	144 (49.0)	1:00	1:00	
30–39	65 (44.2)	118 (40.1)	2.69 (1.61, 4.34)	2.30 (1.21, 4.68)	0.001**
40–49	52 (35.4)	32 (10.9)	7.80 (4.32, 14.07)	4.40 (1.97, 10.12)	0.001**
Educational status					
No formal education	65 (44.2)	209 (71.1)	1.00	1.00	
Grade 1–12	49 (33.3)	44 (15.0)	3.50 (2.18, 5.86)	4.40 (2.18, 8.87)	0.001**
Diploma and above	33 (22.4)	41 (13.9)	2.50 (1.51, 4.42)	2.00 (1.05, 4.59)	0.034*
Residence					
Urban	65 (44.2)	32 (10.9)	6.50 (3.97, 10.60)	2.60 (1.36, 5.21)	0.004*
Rural	82 (55.8)	262 (89.1)	1.00	1.00	
Ever give birth					
Yes	128 (87.1)	127 (43.2)	8.80 (5.19, 15.11)	9.40 (4.92, 18.26)	0.001**
No	19 (12.9)	167 (56.8)	1.00	1.00	
No of sexual partner					
≥ 2	115 (78.2)	135 (45.9)	4.20 (2.68, 6.66)	2.80 (1.60, 5.03)	0.001**
1	32 (21.8)	159 (54.1)	1.00	1.00	
Does your husband have another partner					
Yes	79 (53.7)	66 (22.4)	4.01 (2.62, 6.13)	1.68 (0.95, 2.99)	0.074
No	68 (46.3)	228 (77.6)	1.00		
Attitude					
Negative attitude	58 (39.5)	161 (54.8)	1.00	1.00	
Positive attitude	89 (60.5)	133 (45.2)	0.53 (0.36, 0.80)	2.00 (1.20, 3.70)	0.009*
Knowledge on CCA					
Good knowledge	106 (72.1)	111 (37.8)	4.20 (2.77, 6.55)	3.60 (2.07, 6.43)	0.001**
Poor knowledge	41 (27.9)	183 (62.2)	1.00	1.00	

*p value < 0.05 and **p value < 0.001

## Discussion

Cervical cancer is one of the public health challenges, especially in developing countries. Primary prevention of cervical cancer is a far more feasible approach than secondary or tertiary management of cervical cancer. Identifying the determinants and designing and applying respective strategies are among such cost-effective approaches [37, 38]. In this regard, the current study revealed that the age group of 30–39 and 40–49 years, having multiple sexual partners, ever gave birth, urban residence, educational status and positive attitude towards cervical cancer screening services, and good knowledge towards cervical cancer were predictors of utilization of cervical cancer screening services.

In this study, in the age group of 30–39 and 40–49 years was significantly associated with utilization of cervical cancer screening services as compared to young women (21–29 years). This finding is consistent with the study conducted in Debre Markos, Mekelle, and Addis Ababa, Ethiopia [34, 38, 39]. It was also similar to the study findings in Debre Markos, Mekelle, age groups of 40–49 years had significantly higher odds of being screened [4, 33]. Besides, a study finding from Kenya, also indicated that women in the age groups of 40–49 years had significantly higher odds of being screened for cervical cancer [40]. Similarly, a study conducted in Addis Ababa, reported that willingness and acceptance of screening were higher among women in the age groups of 40–49, 50–59, and > 60 years as compared to those below 29 years[19]. This might be explained that women in higher age groups think themselves at risk of developing cervical cancer.

This study revealed that women with multiple sexual partners were 2.8 times more likely to utilize cervical cancer screening services. This is consistent with a study conducted in Debre Markos; which reported that women with a history of multiple sexual partners were 1.64 times more likely to undergo screening as compared to those who have no history of multiple sexual partners [34]. Another study conducted in Kenya showed that women with more than one- sexual partner were found to be two times more likely to utilize cervical cancer screening services compared to those with single sexual partner [40]. A study conducted in Yirgalem General Hospital, Ethiopia showed that having multiple sexual partners is significantly associated with knowledge of cervical cancer [41]. This can be explained by the fact that women with many sexual partners may perceive themselves to be more at risk for cervical cancer than others. Evidence supplemented that there is a positive association between multiple sexual partners and the development of the precancerous cervical lesion; thus, due to the sexually transmitted nature of HPV, perhaps reflecting a greater likelihood of utilization of screening services.

A positive attitude towards cervical cancer screening was significantly associated with cervical cancer screening services utilization. This finding is supplemented with studies conducted in Debre Markos, and Mekelle, Ethiopia which indicate that the odds of getting screened for cervical cancer were 3.4 times higher among women who had positive attitudes than those who had negative attitudes [33, 34]. Respondents who had negative attitude had 63% lesser odds of being screened compared to those who had positive attitudes towards screening in Nigeria [42]. Those studies were consistent with this study's finding in wolaita positive attitude were 4.8 times more likely to utilize cervical cancer screening than the counterpart [4]. This could be explained that a positive attitude is a precondition to accomplishing a certain task.

Women who had a degree/diploma level of education were 2.0 times more likely to utilize cervical cancer screening services compared with those who did not attend formal education. This is consistent with studies conducted in Dessie referral hospital, Debre Markos, Amhara region, and Addis Ababa, Ethiopia [19, 34, 39]. This finding is also consistent with studies conducted in Kenya, and Swedish which indicated that women who have educated were more likely to utilize cervical cancer screening services than those who had no formal education [40, 43]. This study also supplemented with a study conducted in Yirgalem general hospital, Ethiopia showed that women in secondary education level and degree and above levels were five times and seven times more likely to utilize cervical cancer screening services respectively than those who had no formal education [41]. This might be explained that educated women may have more knowledge of cervical cancer and screening services, and also have more information about cervical cancer screening services. As result, they might develop favorable attitudes and interest to get screened for CCA.

Moreover, in this study women who ever gave birth was nine times more likely to utilize cervical cancer screening services. This is similar to a study conducted at BahirDar which showed that multiparous women were 3 times more likely to utilize the services as compared to those with zero parity [37]. A study from Thailand, and Tanzania revealed a significant relationship between the number of children and cervical cancer screening utilization [44, 45]. This is in line with a study conducted at Yirgalem general hospital, Ethiopia which showed that multipara women were 3.4 times more likely to utilize cervical cancer screening services than their counterparts [41]. This might be explained by the fact that previous pregnancies of a woman may expose her to receive health education and particularly on sexual and reproductive health compared to those with no previous pregnancies.

It is found that women living in urban residences were highly utilizing cervical cancer screening services with (AOR = 2.6, 95% CI 1.36, 5.21). This is consistent with a study conducted in Addis Ababa, Ethiopia which showed that women living in urban were 2 times more likely to utilize cervical cancer screening services than their counterparts [19]. This is in agreement with a study conducted in Kenya, and India which showed that women living in urban were 3.4 times more likely to utilize cervical cancer screening than those who were living in rural area [40, 46]. This might be due to the better availability of health facilities, closer to information, and accessibility of screening services in urban than rural areas.

Good knowledge of cervical cancer was significantly associated with CCA screening service utilization with (AOR = 3.6, 95% CI 2.07, 6.43). This is consistent with studies conducted at Debre Markos and Mekelle, Ethiopia which showed that women who had good knowledge of cervical cancer were 4 and 2.3 times more likely to utilize cervical cancer screening services than their counterparts [30, 41]. However, this finding is different from a study conducted in Tanzania which reported that women who had good knowledge were 3.3 times more likely to utilize cervical cancer screening services than those who had poor knowledge [45]. This might be due to having good knowledge about cervical cancer and its screening may interest in the utilization of cervical cancer screening services.

### Implication for practice

In this study findings, as the determinants for cervical cancer screening service utilization were knowledge and attitude of women towards cervical cancer and its screening, this calls the healthcare authority should create awareness through information, education and communication (IEC), and behavioral change communication (BCC) through health information communication on usage of cervical cancer screening. It is hoped that this will also improve the knowledge and attitude of women. Policymakers like the Ethiopian Ministry of Health (MoH) should ensure that continuing education programs on the use of cervical cancer screening for the prevention of invasive cancer.

### Strengths and limitations of the study

The authors would like to recommend that readers shall interpret certain findings of this study with precautions as recall and social desirability biases might be committed. However, this multi-center study provides valuable information about women’s cervical cancer screening service utilization determinants.

## Conclusion

In this study, women in the age group of 30–39 and 40–49 years, educational status (completed secondary, diploma and above), urban residence, having multiple sexual partners, and ever giving birth were determinant factors to utilize cervical cancer screening services. Good knowledge about cervical cancer screening and a positive attitude towards cervical cancer screening were significantly associated with the uptake of cervical cancer screening services. Results should be considered when developing and implementing strategies to improve CCA screening service utilization in the region.

## Data Availability

The datasets used and/or analyzed during the current study are available from the corresponding author upon reasonable request.

## Abbreviations

CCA: Cervical cancer
CIN: Cervical intraepithelial Neoplasia
HPV: Human papillomavirus
SPSS: Statistical package for social science
STD: Sexually transmitted disease
STI: Sexual transmitted infection
VIA: Visual inspection with acetic acid
WHO: World health organization
AOR: Adjusted odds ratio
COR: Crud odds ratio
CI: Confidence interval
BSC: Bachelor of Science
